# Titanium Ions Promote Exogenous Calcium-Dependent Calcium Influx in Activated Jurkat T Cells: A Possible Mechanism to Explain Its Immunostimulatory Properties

**DOI:** 10.1155/2018/3286905

**Published:** 2018-11-18

**Authors:** Jing Chen, Qiuying Li, Zhenhua Pang, Ming Gong, Li Tang

**Affiliations:** ^1^Department of Implant Dentistry, College of Stomatology, Guangxi Medical University, Nanning 530021, China; ^2^Department of Stomatology, Ruikang Hospital Affiliated to Guangxi University of Chinese Medicine, Nanning 530021, China

## Abstract

Titanium and its alloys have been widely used in dental and orthopedic implants. Owing to the biotribocorrosion behavior of implants in simulated oral environment, Ti(IV) ions could be released into surrounding tissues. Current studies have found that Ti(IV) ions could affect the biological activities of immune cells in adjacent tissues and subsequently jeopardize the long-term performance of implant prostheses. However, the potential mechanism underlying its immunomodulatory properties remains unclear. Calcium signaling has been confirmed to be involved in regulation of lymphocyte immune function. Therefore, we hypothesize that Ti(IV) ions modulated T cell function through the change of intracellular calcium concentrations. This study is aimed at exploring the role of intracellular calcium responses in the modulatory effect of Ti(IV) ions on unactivated and phytohemagglutinin-activated Jurkat T cells. Here, we confirmed that Ti(IV) ions within a certain concentration range induced CD69 expression on both unactivated and activated T cells in our study. Additionally, the combined stimulation with Ti(IV) ions and PHA increased expression of IL-1*β*, TNF-*α*, and RANKL. Furthermore, we found that treatment with Ti(IV) induced a transitory increase in the levels of [Ca^2+^]_i_ in activated Jurkat cells, dependent on the presence of exogenous calcium. Treatment with different doses of Ti(IV) for 24 h significantly increased the levels of [Ca^2+^]_i_ in the activated Jurkat cells in a dose-dependent manner, but had little effect in the unactivated cells. Treatment with Ti(IV) did not significantly affect the PLC*γ*1 activation and inositol-1,4,5-trisphosphate (IP_3_) secretion in Jurkat cells. Taken together, these data indicated that Ti(IV) enhanced calcium influx during the T cell activation, independent of IP_3_-mediated intracellular calcium release. Our work provides insights into the mechanism involved in the regulation of lymphocyte behaviors under the effect of Ti(IV) ions, which may help to develop therapeutic strategies for dental implant failures.

## 1. Introduction

The titanium implant, as a primary component of the dental implant system, was designed to be durable with a high wear resistance, targeting improvement of masticatory function for patients suffering tooth loss. However, growing evidence suggests that such implants can release wear particles and metal ions into surrounding tissues due to a biotribocorrosion process [1–3]. Metal ions released from implants can affect biological activities such as immune functions and have been reported to lead to the premature failure of metallic implants [4, 5].

Previous researches have shown that the titanium ions can induce the activation of monocytes/macrophages, neutrophils, and T lymphocytes and stimulate the production of proinflammatory and bone remodeling cytokines [6–8]. Treatment with Ti(IV) can enhance dendritic cell-mediated T cell responses, particularly for proinflammatory Th1 response [9]. More interestingly, previous in vivo studies have shown that T lymphocytes in the peri-implant microenvironment exhibit stronger activation and proliferation in patients with loosen implants [10, 11]. Given that activated T cells can secrete inflammatory cytokines, which can result in inflammation-associated bone loss [12, 13], further understanding the precise role of Ti(IV) in regulating T cell activation will be of significance.

During the T cell activation, engagement of T cell receptor (TCR) by antigen determinant initiates T cell activation by increasing the levels of intracellular calcium ([Ca^2+^]_i_) [14]. The increased levels of [Ca^2+^]_i_ can come from intracellular pool, which is positively controlled by inositol-1,4,5-trisphosphate (IP_3_). Furthermore, the increased levels of [Ca^2+^]_i_ can also stem from enhanced calcium influx [15]. The increased levels of [Ca^2+^]_i_ are crucial for subsequent function of activated T cells, such as functional differentiation and effector cytokine production [16, 17]. However, there is no information on whether and how Ti(IV) can modulate the levels of [Ca^2+^]_i_ in T cells.

In this study, we address a potential effect, mediated by Ca^2+^ signaling, of titanium ions on Jurkat T lymphocytes and investigate the expression levels of proinflammatory and bone remodeling cytokines.

## 2. Materials and Methods

### 2.1. Cell Culture

Human acute T lymphocyte leukemia Jurkat (Clone E6–1) cells were obtained from the Cell Resource Center of Chinese Academy of Medical Sciences (Beijing, China). The cells were cultured in RPMI 1640 medium supplemented with 10% fetal bovine serum (FBS) and 100 units/ml of penicillin and 100 *μ*g/ml of streptomycin at 37°C in a 5% CO_2_ atmosphere. The cells (1 × 10^6^ cells/well) were stimulated with, or without, 5 *μ*g/ml PHA (Sigma, St. Louis, USA) in 6-well plates for 24 h and treated with different reagents for subsequent experiments.

### 2.2. Flow Cytometric Detection of CD69 Expression

For analysis of CD69 expression on the cell surface, Jurkat T cells were incubated in 6-well plates as described above and then treated with Ti(IV) chloride (Sigma, St. Louis, USA) at different concentrations (5, 25, 50, or 100 *μ*M). The purity of Ti(IV) chloride was >99.995% as determined by trace metal analysis, and the Ti(IV) chloride solution did not contain detectable bacterial endotoxin lipopolysaccharide (LPS), determined using the ToxinSensorTM Chromogenic LAL Endotoxin Assay Kit (GenScript, NJ, USA), according to the manufacturer's instruction. After 24 h of incubation, cells were harvested and then labeled with 5 *μ*l anti-CD69-PE antibody (eBioscience, CA, USA) for 30 min at room temperature. The percentage of T cell-expressing CD69 was determined using flow cytometer (Becton Dickinson, NJ, USA).

### 2.3. ELISA for Cytokine Determination

Cells were cultured with Ti(IV) ions (5, 25, 50, or 100 *μ*M) alone or in combination with 5 *μ*g/ml of PHA for 24 h. The cell supernatants were then collected and assayed for IL-1*β*, TNF-*α*, and RANKL concentrations by the enzyme-linked immunosorbent assay (Cusabio Biotech, China).

### 2.4. Real-Time Measurement of Ca^2+^ Transients with Laser Scanning Confocal Microscopy

The unactivated and activated Jurkat cells were loaded with 5 *μ*M acetoxymethyl ester of fluo-3 (fluo-3/AM) in the presence of 0.02% Pluronic F-127 (Biotium, Hayward, USA) for 45 min at 37°C in 5% CO_2_. The cells were washed three times with 10 mM HEPES buffer to remove free dye and continually cultured for additional 30 min to complete deesterification. Before imaging, the cells were suspended in calcium-containing solution (145 mM NaCl, 2.8 mM KCl, 2 mM MgCl_2_, 2 mM CaCl_2,_ 10 mM glucose, and 10 mM HEPES) or calcium free solution (145 mM NaCl, 2.8 mM KCl, 2 mM MgCl_2_, 1 mM EGTA, 10 mM glucose, and 10 mM HEPES). The cell suspension (50 cells/*μ*l) was plated in the poly-L-lysine- (Sigma) coated 20 mm glass-bottom culture dishes (MatTek, Ashland, USA). The cells were treated with Ti(IV) chloride (Sigma, St. Louis, USA) at different concentrations (5, 25, 50, or 100 *μ*M) and observed under a confocal laser scanning microscope (Nikon, Japan, Ex/Em: 488 nm/526 nm). The fluorescent images of appropriate T cells were captured at 526 nm and scanned every 2 s. The changes in fluorescent signal intensity were recorded as the changes in [Ca^2+^]_i_ following treatment with Ti(IV) ions. The changes in [Ca^2+^]_i_ were calculated by F1/F0 (F0, control; F1, administration of Ti(IV) ions).

### 2.5. Flow Cytometric Analysis of Intracellular Ca^2+^ Concentration

The unactivated and activated Jurkat cells (1 × 10^6^ cells/well) were treated in triplicate with Ti(IV) ions (5, 25, 50, or 100 *μ*M) in 6-well plates in culture medium containing Ca^2+^ (2 mM) or calcium-free medium for 24 h. The cells were harvested and adjusted to 1 × 10^6^ cells/ml. The cells were loaded with 5 *μ*M fluo-3/AM for 45 min in the presence of 0.02% Pluronic F-127. After being washed, the cells were analyzed by flow cytometry in an influx spectrofluorometer (Becton Dickinson, USA) with excitation at 488 nm and emission at 526 nm. The maximal fluorescence (*F*_max_) was determined over 5 min after lysing the cells and subcellular organelles with Triton X-100 (0.2% *v*/*v*, Sigma). The minimum fluorescence (*F*_min_) was determined over a further 5 min following treatment with 10 mM ethylene glycol-bis(beta-aminoethyl ether)-N,N,N′,N′-tetraacetic acid (EGTA). The fluorescent signal values were converted to [Ca^2+^]_i_ using the following equation:

(1) $$\begin{array}{c} Free{\left[Ca^{2+}\right]}_{i}=K_{d}\left[\frac{\left(F-F_{\min}\right)}{\left(\;F_{\max}-F\right)}\right], \end{array}$$

where *K*_d_ (450 nM) is the dissociation constant of the fluo-3/Ca^2+^ complex, *F* is the measured fluorescence intensity, *F*_min_ is the minimum fluorescent signals at a very low Ca^2+^ condition, and *F*_max_ is the fluorescent signals measured at a high Ca^2+^ condition.

### 2.6. Western Blot Analysis of PLC*γ*1 Phosphorylation

Jurkat T cells were stimulated as described above for 24 h, washed with cold PBS, and lysed in RIPA buffer containing 2 mM PMSF for 20 min on ice. After centrifugation and quantification of protein concentrations, the cell lysates (30 *μ*g/lane) were analyzed by sodium dodecyl sulfate polyacrylamide gel electrophoresis (SDS-PAGE) and transferred electrophoretically onto polyvinylidene difluoride (PVDF) membrane. The membranes were blocked with 5% fat-free dry milk in TBST and were probed with rabbit anti-PLC*γ*1, anti-p-PLC*γ*1, or anti-*β*-actin. The bound antibodies were detected with horseradish peroxidase- (HRP-) conjugated secondary antibodies and visualized using the enhanced chemiluminescent reagents (Amersham, Little Chalfont, UK).

### 2.7. Measurement of IP_3_ by ELISA

The levels of IP_3_ in the supernatants of cultured cells were measured by enzyme-linked immunosorbent assay (ELISA). Briefly, the unactivated and activated Jurkat cells (1 × 10^6^ cells/well) were treated in triplicate with, or without, Ti(IV) ions (5, 25, 50, or 100 *μ*M) in 6-well plates for 24 h. Their supernatants were harvested and the levels of IP_3_ in the supernatants of cultured cells were measured by ELISA using the specific kit (Cusabio Biotech, China), according to the manufacturer's protocol. The reactions were measured at 450 nm in the microplate reader (Molecular Devices). The concentrations of IP_3_ were determined by a standard curve established using different concentrations of IP_3_ provided.

### 2.8. Statistical Analysis

All data were present as the mean ± standard deviation (SD). Statistically significant differences between group means were determined by analysis of variance (ANOVA) using the SPSS18.0 program. Post hoc testing was performed for intergroup comparisons using the least significance difference (LSD) test. A *P* value of <0.05 was considered statistically significant.

## 3. Results

### 3.1. Ti(IV) Ions Promote CD69 Expression on Both Unactivated and Activated T Cells

**Figure 1:**
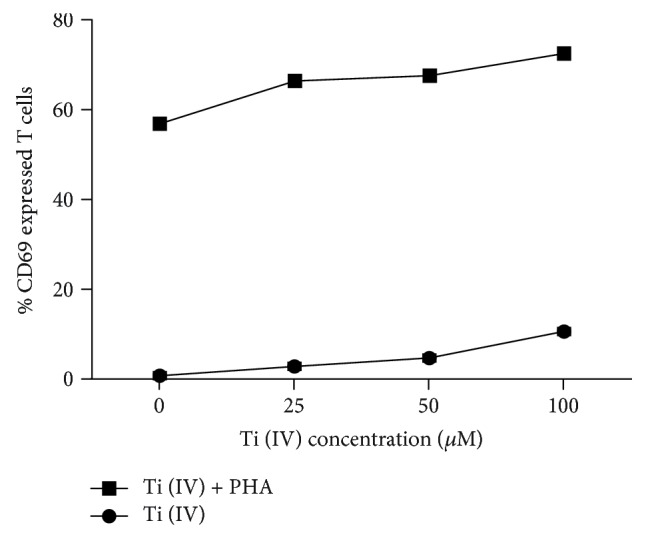
Percentage of CD69-expressed T cells after stimulation with Ti(IV) ions (0, 25, 50, and 100 *μ*M) in the presence or absence of PHA. Data are expressed as the mean ± SD of each group from three independent experiments.

To evaluate the effect of Ti(IV) ions on T cell activation, we assessed the expression of its early marker CD69. Flow cytometry analysis showed that Ti(IV) ions dose-dependently enhanced CD69 expression on both unactivated and PHA-activated T cells (Figure 1). Furthermore, the enhancement effect of Ti(IV) ions on CD69 expression was more significant in activated T cells than that in unactivated cells.

### 3.2. Ti(IV) Ions Increase Expression of IL-1*β*, TNF-*α*, and RANKL in Activated T Cells

**Figure 2:**
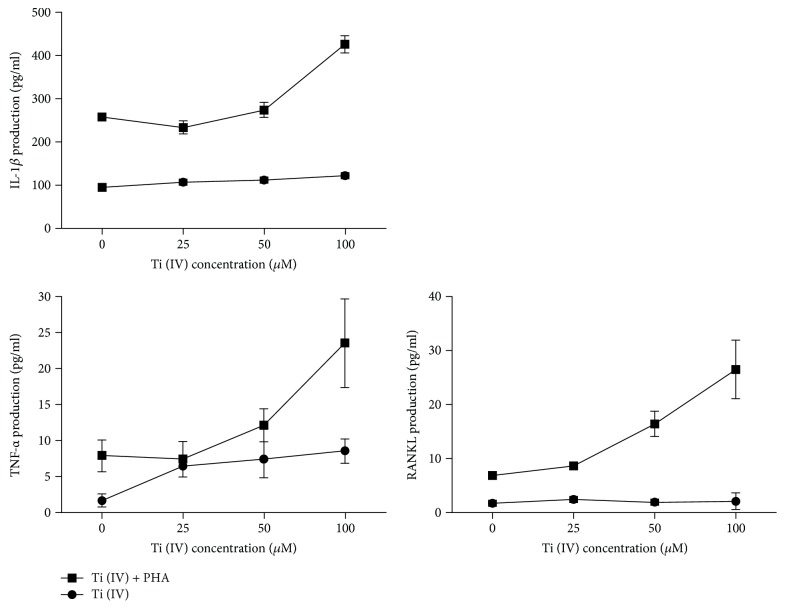
Cytokine production in T cells treated with Ti(IV) ions. Jurkat T cells were treated by 0, 25, 50, and 100 *μ*M of Ti(IV) ions with or without PHA (5 *μ*g/ml) for 24 h. Then, the cell supernatants were collected and IL-1*β*, TNF-*α*, and RANKL concentration was determined by ELISA, respectively. Each point is the mean of three independent experiments.

To examine whether the stimuli from Ti(IV) ions affect the expression of proinflammatory and bone remodeling cytokines, cells were treated with Ti(IV) ions in the presence or absence of PHA for 24 h and then the expression levels of IL-1*β*, TNF-*α*, and RANKL were measured. Compared with unactivated T cells, the expression of IL-1*β*, TNF-*α*, and RANKL in activated T cells was significantly increased by Ti(IV) ions (Figure 2). Furthermore, the stimulatory activities of Ti(IV) ions on this cytokine production were concentration-dependent.

### 3.3. Ti(IV) Ions Induce Transitory Calcium Influx in Activated T Cells

**Figure 3:**
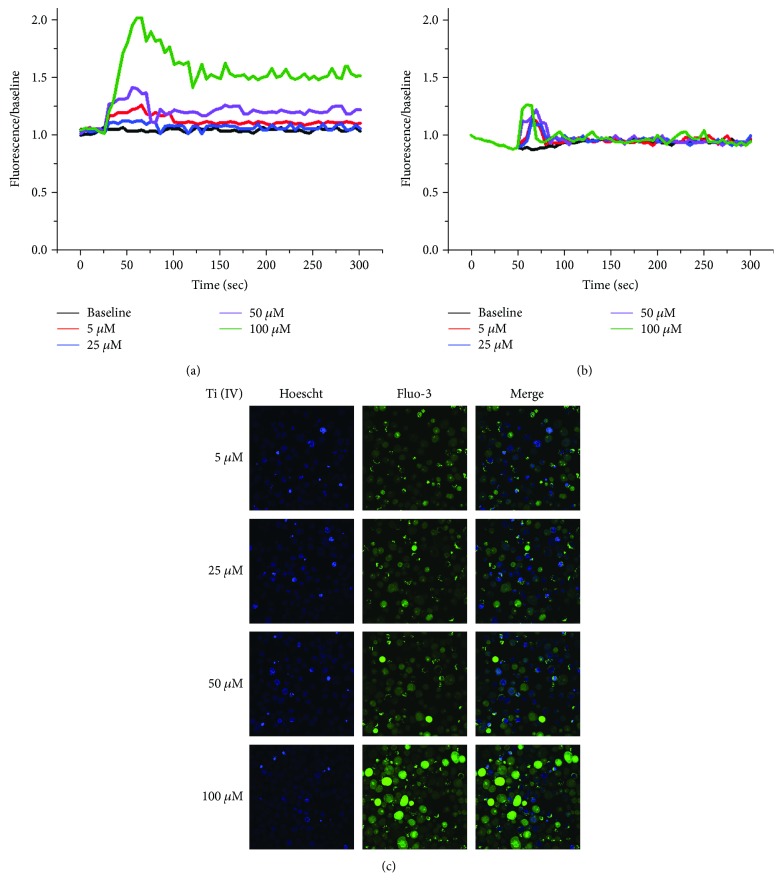
Fluorescence imaging and [Ca^2+^]i analysis of PHA-activated T cells exposed to Ti(IV) ions. Ti(IV) induces transitory calcium influx in activated T cells (a), but has little effect on intracellular calcium release (b). Data are representative images from three separate experiments. (c) Fluorescent images captured by laser scanning microscopy show the activated cells exposed to different concentrations of Ti(IV) ions in the presence of extracellular Ca^2+^.

**Figure 4:**
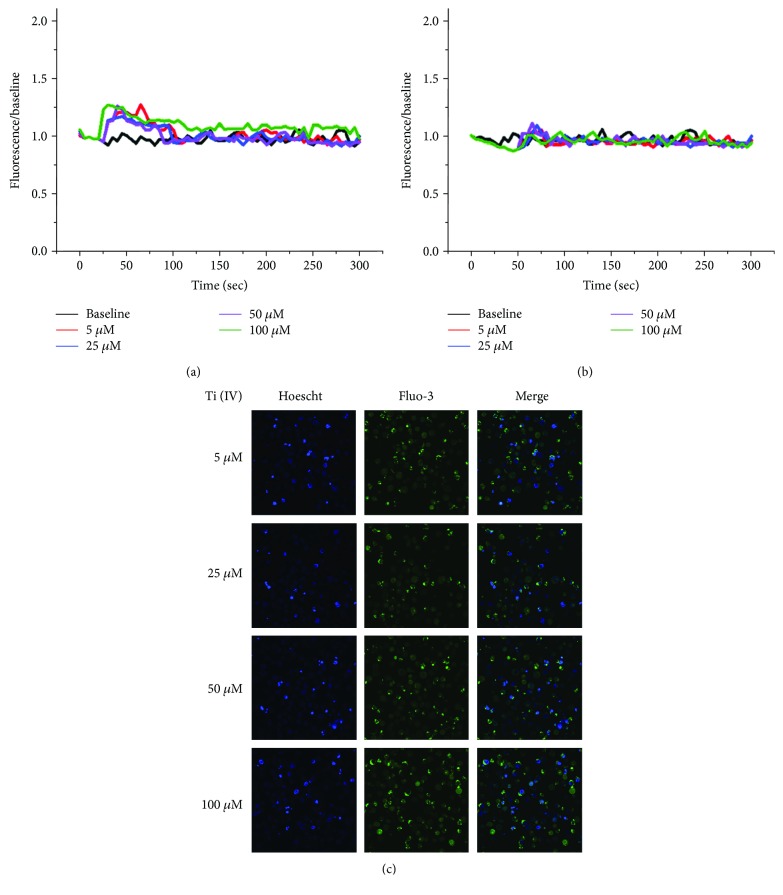
Fluorescence imaging and [Ca^2+^]i analysis of unactivated T cells exposed to Ti(IV) ions. Ti(IV) has non-significant effect on calcium mobilization in unactivated T cells. Data are representative images from three separate experiments. (c) Fluorescent images captured by laser scanning microscopy show the unactivated cells exposed to different concentrations of Ti(IV) ions in the presence of extracellular Ca^2+^.

To test the hypothesis that Ti(IV) ions can enhance T cell response through activating the calcium signaling, we investigated the effect of Ti(IV) ions on the dynamic changes in the levels of [Ca^2+^]i in Jurkat T cells. Jurkat cells were stimulated with, or without, PHA for 24 h. The unactivated and activated cells were treated with different concentrations of Ti(IV) ions in the presence of extracellular Ca^2+^, and the dynamic changes in the levels of [Ca^2+^]i were characterized by laser confocal microscopy. As shown in Figure 3(a), treatment with Ti(IV) induced a transitory increase in [Ca^2+^]_i_ at about 30s post treatment, which declined to moderate levels throughout the observation period and its effects appeared to be dose-dependent in the activated Jurkat cells. In the absence of extracellular Ca^2+^, treatment with Ti(IV) ions resulted in a temporary increase in [Ca^2+^]i, which fell rapidly to near the basal level within 50 s in the activated cells (Figure 3(b). Immunofluorescent images indicated that there were some activated cells with Fluo-3/AM/Ca^2+^ and the fluorescence intensity increased obviously with the changes in the concentration of Ti(IV) ions (Figure 3(c)).In contrast, there were only moderate levels of abrupt increases in [Ca^2+^]_i_ in the unactivated cells at the similar time periods (Figure 4(a)). In the absence of extracellular Ca^2+^, treatment with Ti(IV) ions failed to induce any response in the unactivated cells (Figure 4(b)). It was observed that only a few of unactivated cells displayed fluorescent signals, and no significant change in the fluorescence intensity increased upon Ti(IV) stimulation (Figure 4(c)). Collectively, treatment with Ti(IV) promoted transitory calcium influx in activated T cells.

### 3.4. Treatment with Ti(IV) for a Long Period Promotes Calcium Influx in Jurkat Cells

**Figure 5:**
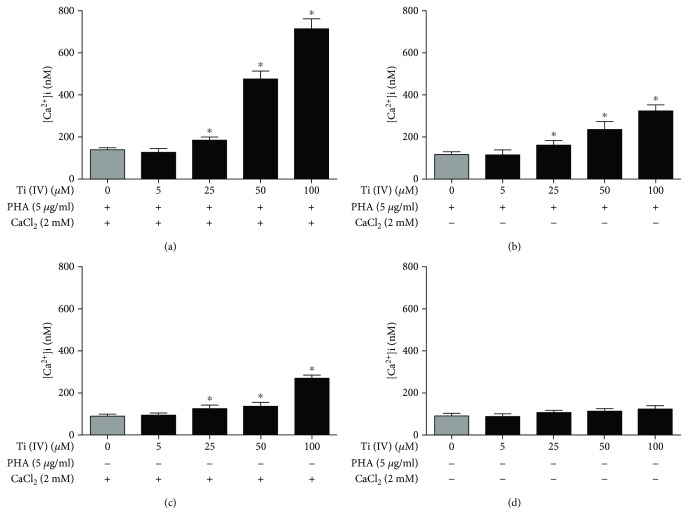
Flow cytometric measurement of calcium influx after long-term treatment with Ti(IV) ions. Jurkat T cells were stimulated with, or without, PHA for 24 h and treated with the indicated doses of Ti(IV) for 24 h in the presence of exogenous calcium or EGTA. (a, b) The levels of [Ca^2+^]_i_ in activated cells. (c, d) The levels of [Ca^2+^]_i_ in unactivated cells. Data are expressed as the mean ± SD of each group from three separate experiments and differences between mean values were assessed by one-way ANOVA. ^∗^*P* < 0.05 indicates a statistically significant difference compared with the control group.

Next, we tested the effect of long-term treatment with Ti(IV) on the levels of [Ca^2+^]_i_ in the unactivated and activated Jurkat cells. The unactivated and activated cells were treated with, or without, 5, 25, 50, or 100 *μ*M Ti(IV) for 24 h in the presence of 2 mM CaCl_2_ or 10 mM EGTA. The levels of [Ca^2+^]_i_ were analyzed by flow cytometry. Treatment with Ti(IV) in the presence of exogenous calcium significantly increased the levels of [Ca^2+^]_i_ in activated cells in a dose-dependent manner, which were dramatically mitigated in the presence of EGTA (Figures 5(a) and 5(b)). In contrast, treatment with Ti(IV) for 24 h in the presence of 2 mM CaCl_2_ resulted in a moderate increase in the levels of [Ca^2+^]_i_ in the unactivated cells, but other experimental conditions did not increase the levels of [Ca^2+^]_i_ in the unactivated cells (Figures 5(c) and 5(d)). Thus, long-term treatment with Ti(IV) in the presence of exogenous calcium promoted calcium influx in Jurkat cells.

### 3.5. Effect of Ti(IV) Ions on PLC*γ*1 Phosphorylation

**Figure 6:**
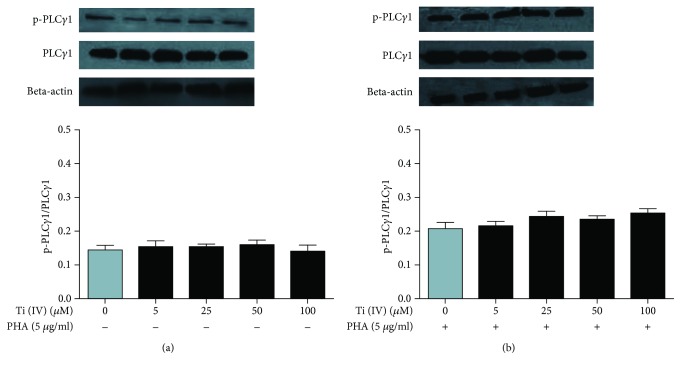
Western blot analysis of PLC*γ*1 phosphorylation. The unactivated and activated cells were treated with Ti(IV) at the indicated doses for 24 h and the levels of PLC*γ*1 expression and phosphorylation in the different groups of cells were determined by Western blot. Data are representative images of each group. (a) The levels of PLC*γ*1 phosphorylation in unactivated cells. (b) The levels of PLC*γ*1 phosphorylation in activated cells.

The phosphorylation of PLC*γ*1 leads to the activation of IP_3_, which is a critical regulator of intracellular calcium release from the calcium pool during the T cell activation [18]. We measured the changes in the levels of PLC*γ*1 phosphorylation in Jurkat cells after exposed to Ti(IV) for 24 h. As shown in Figure 6, treatment with either dose of Ti(IV) did not significantly change the levels of PLC*γ*1 phosphorylation in both unactivated and activated cells regardless of Ti(IV) dosages. Hence, Ti(IV) did not affect the activation of PLC*γ*1 in Jurkat T cells.

### 3.6. Effect of Ti(IV) Ions on IP_3_ Secretion

**Figure 7:**
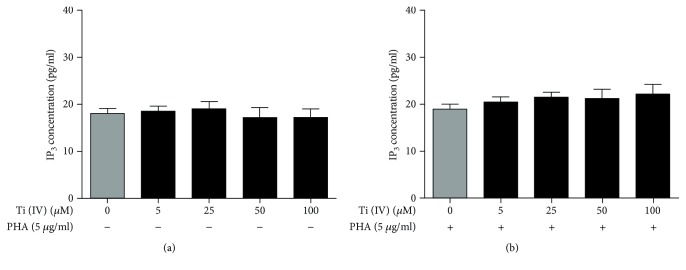
Quantitative analysis of IP_3_ production in Jurkat cells following Ti(IV) treatment. The unactivated and activated cells were treated with Ti(IV) at the indicated doses for 24 h and the supernatants of cultured cells were harvested. The levels of IP_3_ in individual supernatant samples were determined by ELISA. Data were expressed as the mean ± SD of each group from three separate experiments.

Finally, we measured the levels of IP_3_ in the supernatants of cultured Jurkat cells by ELISA following treatment with different concentrations of Ti(IV) for 24 h. In comparison with that in the untreated controls, there was no significant difference in the levels of IP_3_ among the different groups of cells regardless of unactivated and activated Jurkat cells (*P* > 0.05, Figures 7(a) and 7(b)). Therefore, treatment with Ti(IV) did not affect the levels of secreted IP_3_ in the cultured Jurkat cells in vitro.

## 4. Discussion

Depending on the sort of metals, concentration, exposure time, and the environment of affected immune cells, metal ions may either suppress or stimulate immune cell activity [19–21]. For example, Ti(IV) ions inhibit T and B cell activation in vitro [22], but stimulate monocytes/splenocytes to secrete higher concentrations of IL-1*β*, TNF-*α*, and IFN-*γ* in the presence of other stimulus [23, 24]. Therefore, it is important to use resting or mitogen-activated T cells when exploring the mechanism underlying the immunomodulatory effect of Ti(IV) ions.

Jurkat cells, a human T cell leukemia cell line, were used to explore the effect and underlying mechanism of Ti(IV) ions on the biological behavior of T cells. In this study, we observed that Ti(IV) ions promote T cell activation and subsequent expression of proinflammatory cytokines, whereas the stimulatory effect was more pronounced in activated T cells. These results are compatible with data reported by Cadosch et al. [8] and the action mechanisms may be through interfering with signaling pathways involved in T cell activation and cytokine production.

Given that increased levels of intracellular calcium are necessary for activation-related downstream signaling and T cell function [16], it is possible that the effects of Ti(IV) ions may target the calcium signaling pathways in mitogen-activated T cells. In this work, we examined the effect of treatment with different doses of Ti(IV) on the dynamic changes in the levels of intracellular calcium in unactivated and PHA-activated Jurkat T cells. Our results indicate that Ti(IV) ions increased the levels of intracellular calcium in both the activated and unactivated T cells, but the effect was more significant in activated T cells. When comparing Ti(IV)-induced changes in [Ca^2+^]_i_, expression levels of CD69, IL-1*β*, and TNF-*α* expression, there was a very clear correlation between these responses. From the present results, we suggest that the stimulatory mechanisms of Ti(IV) ions on activated T cells activation and inflammatory cytokine expression, at least in part, are related to the increase of [Ca^2+^]_i_ in the cells.

The elevated intracellular calcium can be the consequence of Ca^2+^ release from intracellular calcium pools or/and increased Ca^2+^ influx from extracellular environment [25]. We found that treatment with any of the doses of Ti(IV) in calcium-contained medium significantly increased the levels of intracellular calcium in activated Jurkat cells in a dose-dependent manner. However, treatment with Ti(IV) in the presence of EGTA did not significantly increase the levels of intracellular calcium in both unactivated and activated Jurkat cells. Such data indicated that increased levels of intracellular calcium induced by Ti(IV) may depend on exogenous calcium. It is well known that activation-related signals can activate PLC, which hydrolyses PIP_2_ to release IP_3_ and release calcium from intracellular pool in activated T cells [26]. Inositol (1,4,5)-trisphosphate receptors (IP_3_Rs) respond to this elevation of IP_3_ by releasing Ca^2+^, which can lead to the initiation of calcium signaling [27]. We found that treatment with Ti(IV) ions did not change the PLC*γ*1 phosphorylation and IP_3_ levels in the supernatants of cultured unactivated and activated Jurkat T cells. These results further indicated that treatment with Ti(IV) ions has little effect on early IP_3_-mediated calcium release and the elevated levels of intracellular calcium by Ti(IV) depended on calcium influx in activated Jurkat T cells.

Unlike other many types of cells, Ca^2+^ release in activated T cells makes a relatively smaller contribution to the levels of [Ca^2+^]_i_ and downstream signaling than the exogenous calcium-dependent Ca^2+^ influx [14, 15]. Our data revealed that Ti(IV) ions promoted calcium influx in activated Jurkat cells in a dose-dependent manner. Thus, it appears that Ca^2+^ influx, rather than the release of Ca^2+^ from IP_3_-sensitive pool, may be crucial for the modulatory effects of Ti(IV) ions in activated T cells via the calcium signaling pathway. Calcium influx in T cells depends on a complex interplay among intracellular Ca^2+^ stores, plasma membrane calcium channels, and mitochondrion, which determine the levels of [Ca^2+^]_i_ [28, 29]. Prolonged Ca^2+^ influx through calcium channels is crucial in activating the nuclear factor of activated T cells (NFAT), a Ca^2+^-sensitive transcription factor responsible for regulation of T cell activation and cytokine expression [30, 31]. It was notable that even though Ti(IV) ions alone induced transitory calcium influx, the levels of [Ca^2+^]_i_ at 24 h posttreatment were not significant, which might not be enough to maintain NFAT in the nucleus. Evidence suggests that transient activation of NFAT was not sufficient to induce the expression of several cytokines [32]. These findings may explain why treatment with Ti(IV) ions increases the production of receptor activator of nuclear factor *κ*B ligand (RANKL) in PHA-stimulated T cells, but the effect was not significant in unactivated T cells.

There are hints of evidence supporting the idea that the species Fe(C)Ti(N)-TF might provide a route for Ti(IV) entry into cells via the transferrin receptors after the release of metal ions from its implants [33, 34]. Additionally, Ti(IV) has a strong affinity for phosphorous-containing molecules [35]. It is possible that Ti(IV) may bind to phosphorylated intracellular proteins, which often correspond to active functional states of enzymes or signaling proteins and interfere with signaling pathways. Further studies are necessary to explore the molecular mechanisms underlying the action of Ti(IV) in promoting Ca^2+^ influx in activated T cells.

Taken together, the present study suggests that small amounts of titanium ions released from metal implants may enhance inflammatory T cell responses by increasing calcium influx in activated T cells. These findings from this study are significant in understanding the immunostimulatory effect of Ti(IV) ions on T lymphocytes and its underlying mechanism, especially in the presence of other inflammatory mediators, and hence provide new insights into the process and therapeutic strategies of inflammatory osteolysis.

## Data Availability

The data used to support the findings of this study are available from the corresponding author upon request.
